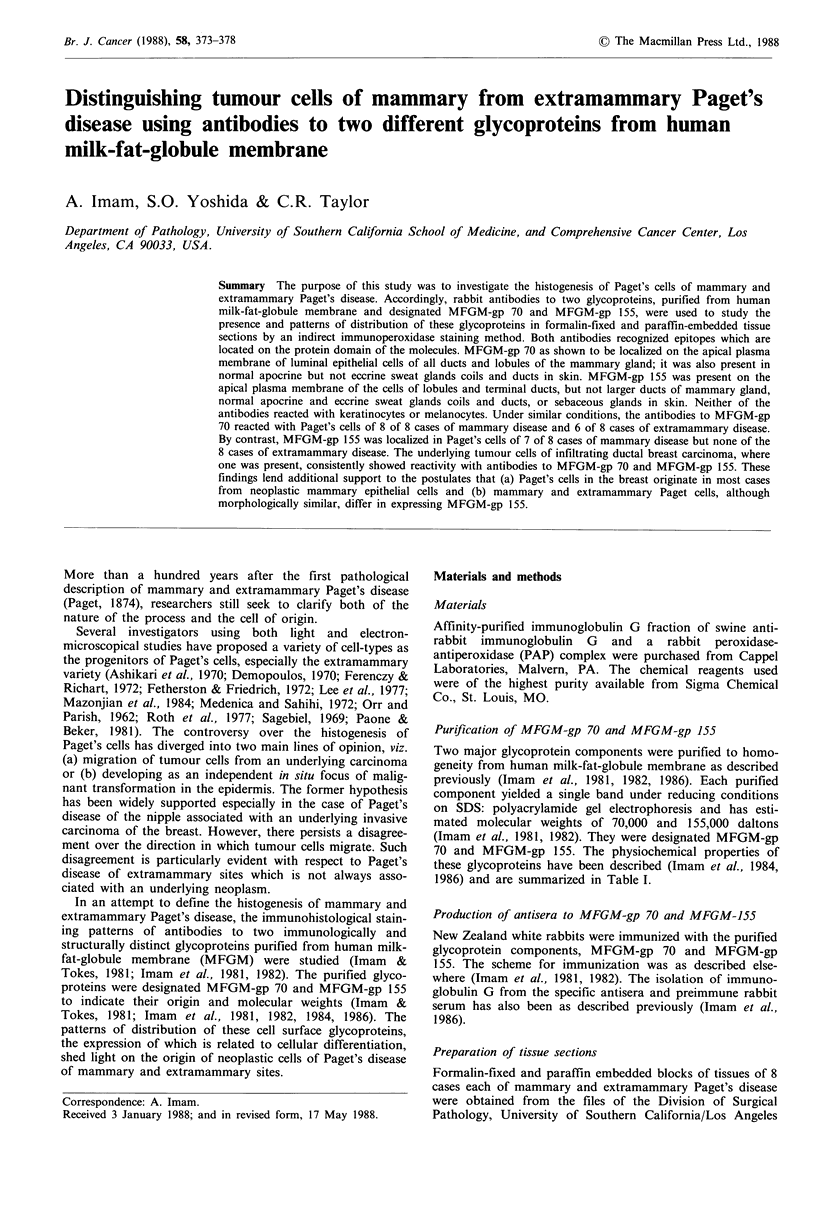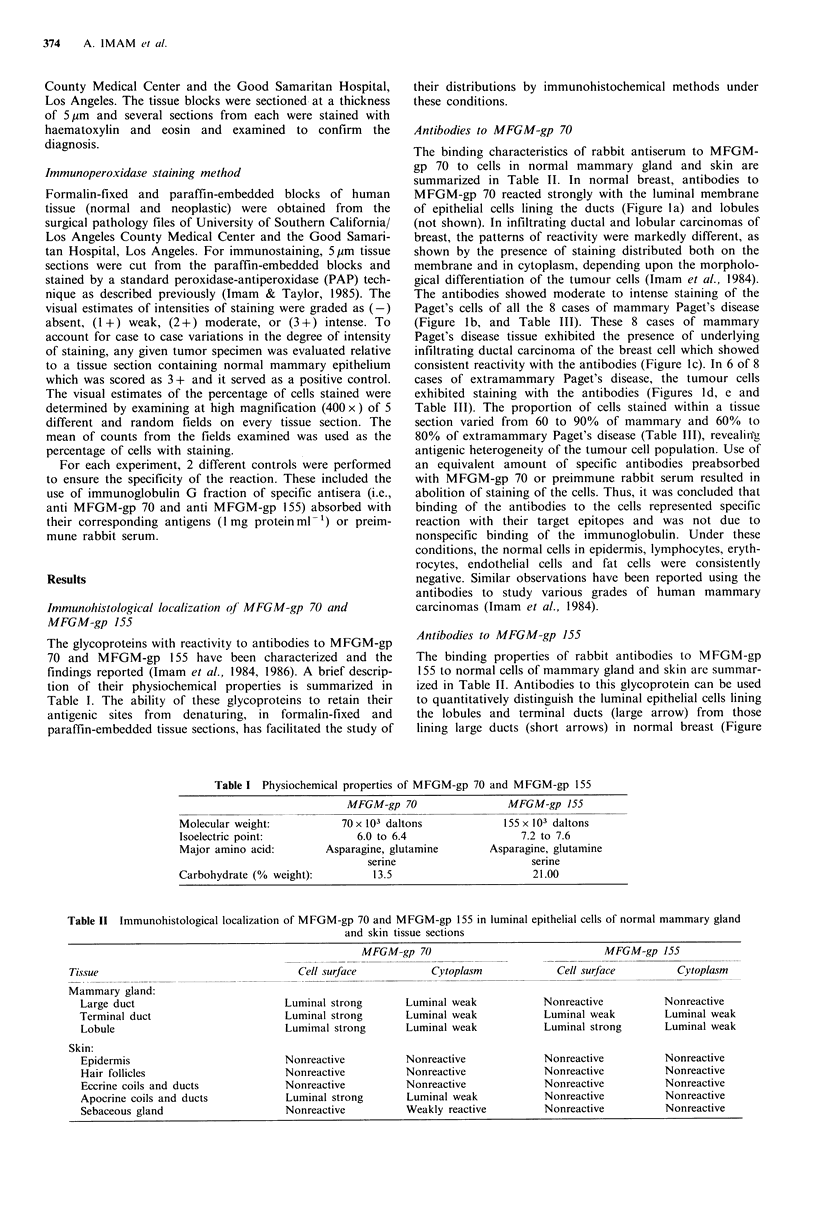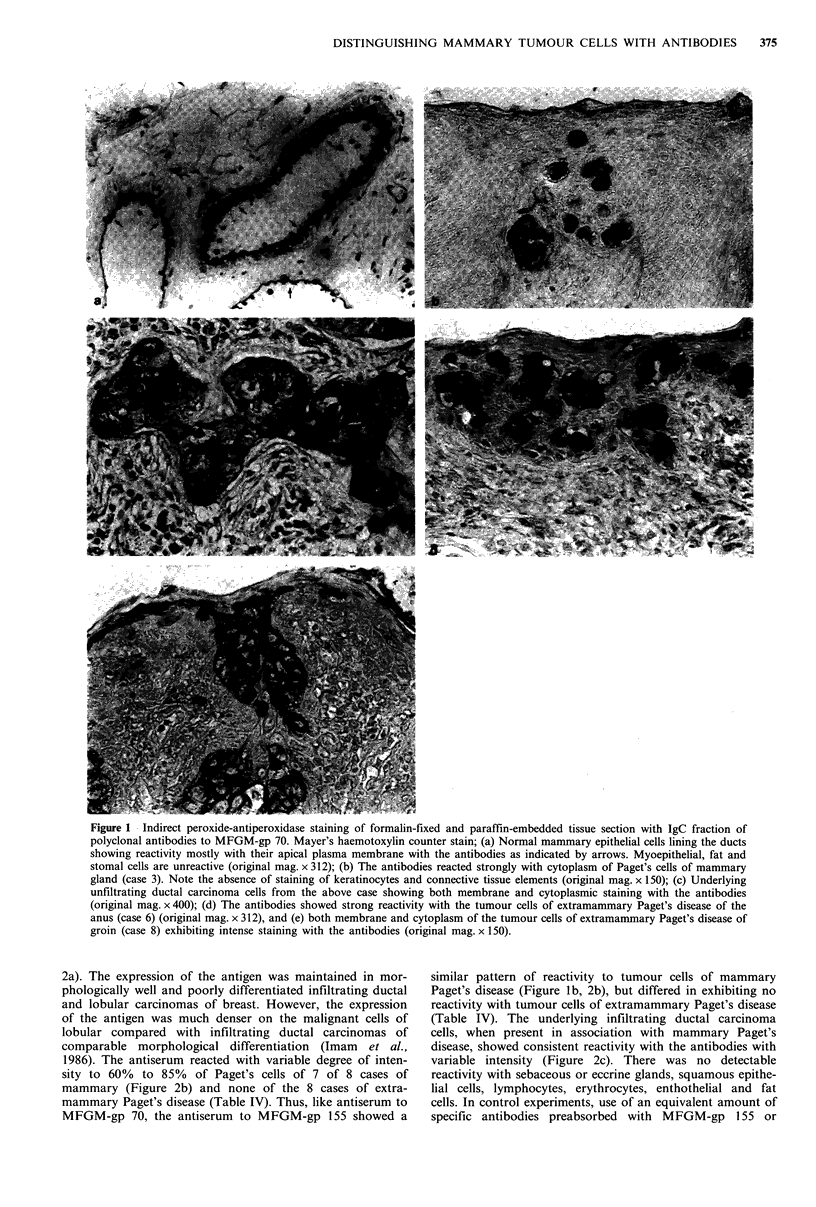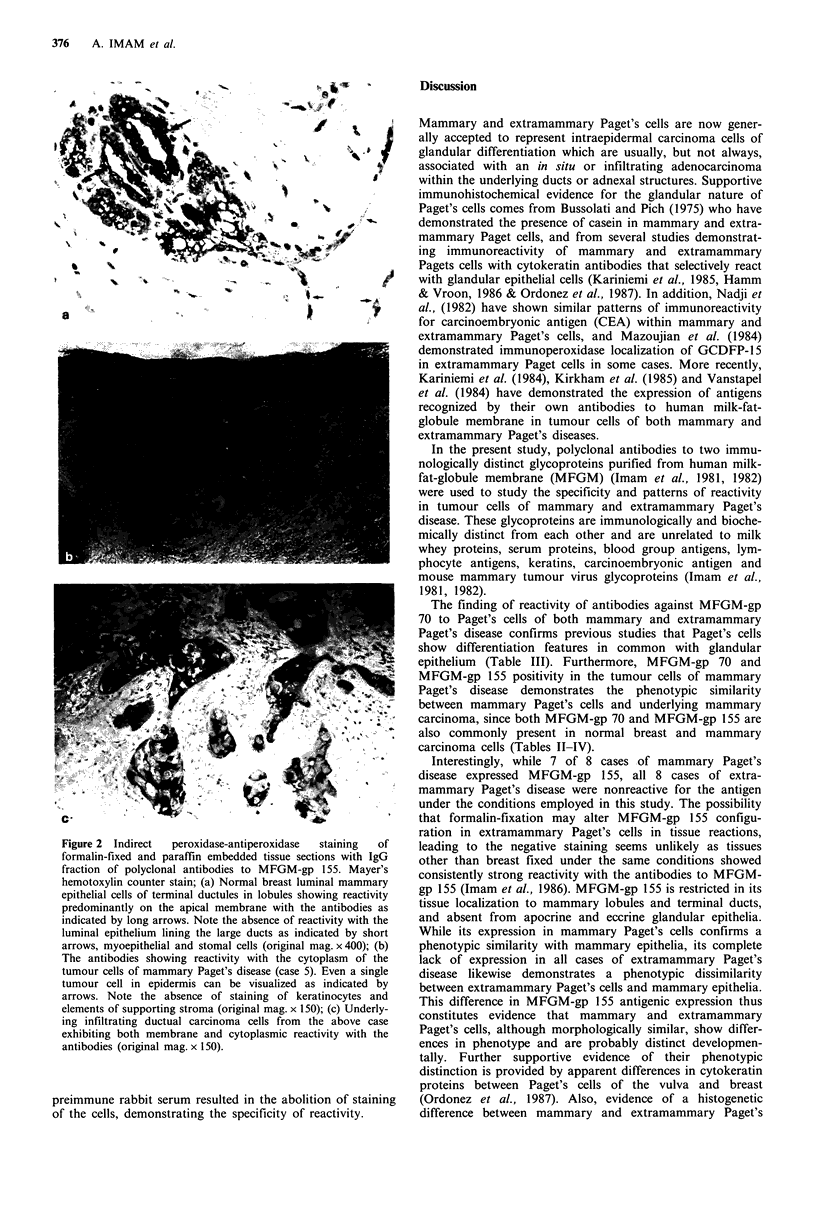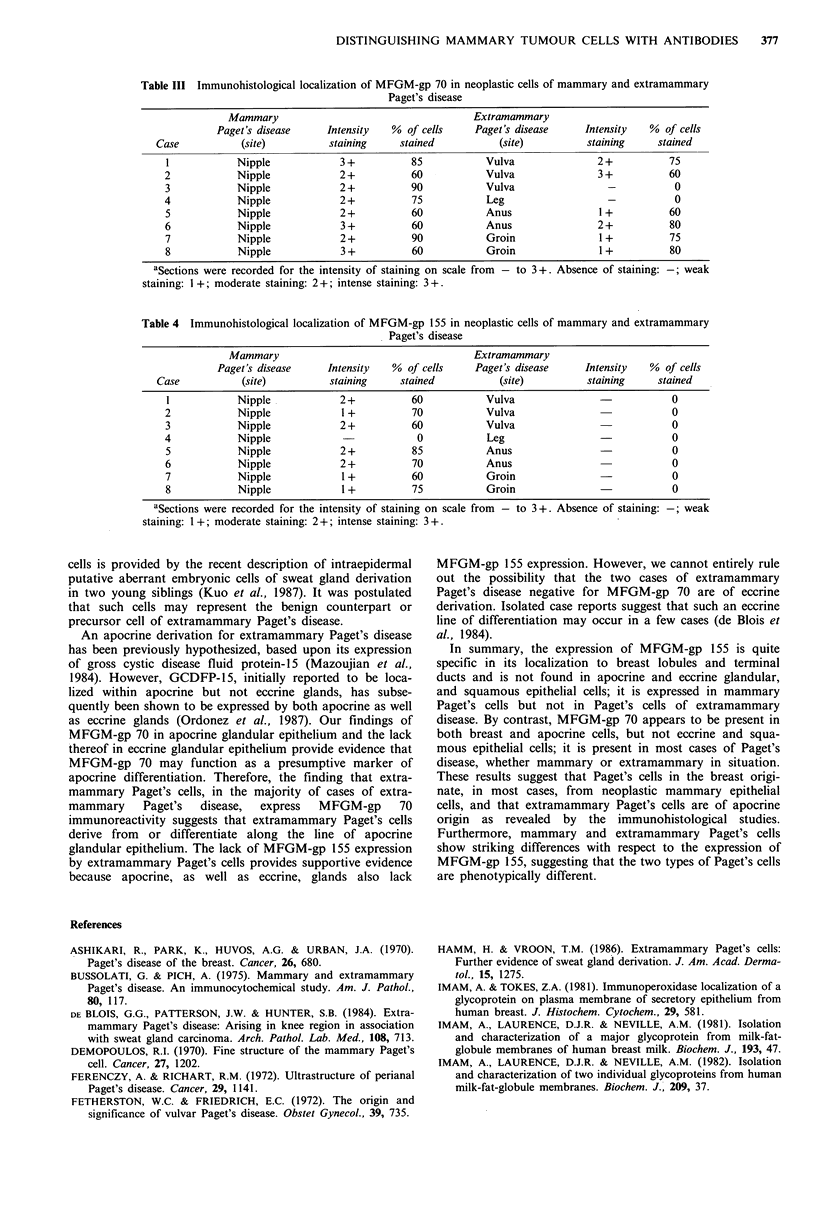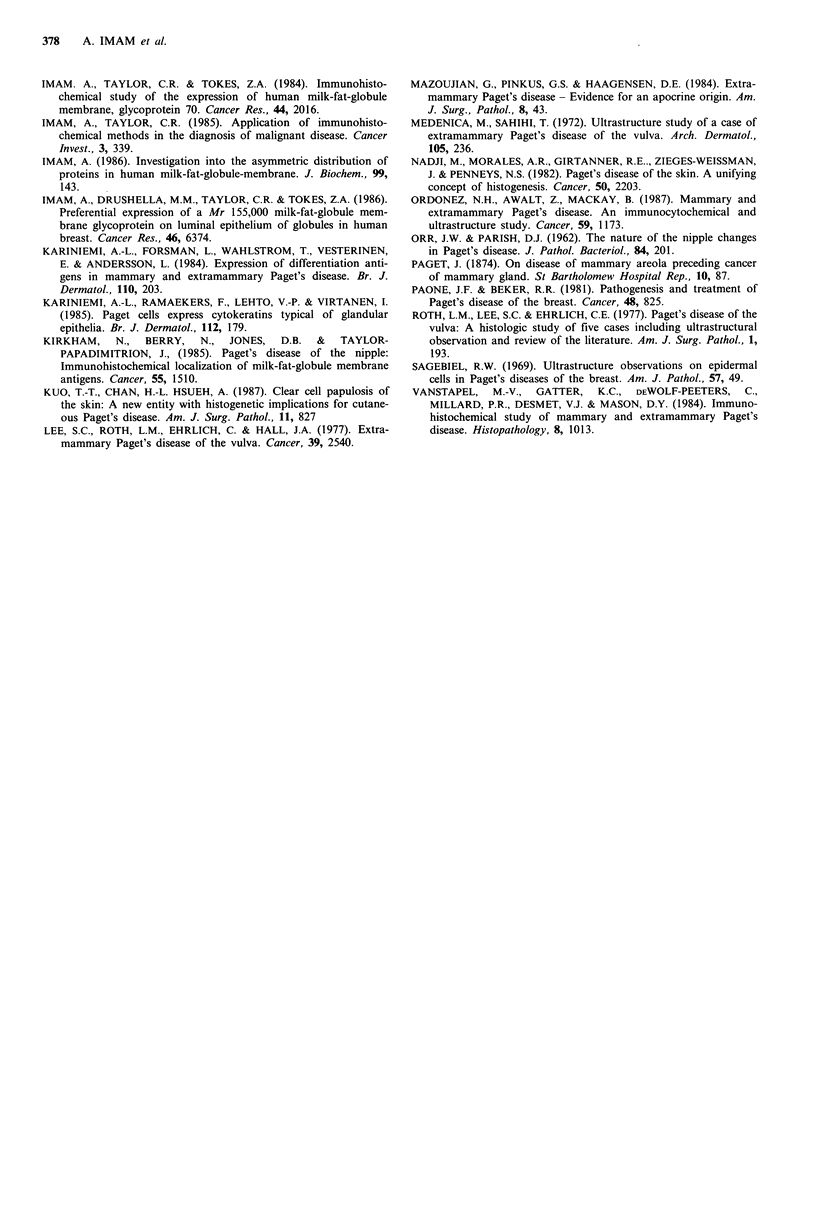# Distinguishing tumour cells of mammary from extramammary Paget's disease using antibodies to two different glycoproteins from human milk-fat-globule membrane.

**DOI:** 10.1038/bjc.1988.222

**Published:** 1988-09

**Authors:** A. Imam, S. O. Yoshida, C. R. Taylor

**Affiliations:** Department of Pathology, University of Southern California School of Medicine, Los Angeles 90033.

## Abstract

**Images:**


Br. J. Cancer (1988), 58, 373–378 © The Macmillan Press Ltd., 1988

### Distinguishing tumour cells of mammary from extramammary Paget's disease using antibodies to two different glycoproteins from human milk-fat-globule membrane

A. Imam, S.O. Yoshida & C.R. Taylor

*Department of Pathology, University of Southern California School of Medicine, and Comprehensive Cancer Center, Los Angeles, CA 90033, USA.*

**Summary** The purpose of this study was to investigate the histogenesis of Paget's cells of mammary and extramammary Paget's disease. Accordingly, rabbit antibodies to two glycoproteins, purified from human milk-fat-globule membrane and designated MFGM-gp 70 and MFGM-gp 155, were used to study the presence and patterns of distribution of these glycoproteins in formalin-fixed and paraffin-embedded tissue sections by an indirect immunoperoxidase staining method. Both antibodies recognized epitopes which are located on the protein domain of the molecules. MFGM-gp 70 as shown to be localized on the apical plasma membrane of luminal epithelial cells of all ducts and lobules of the mammary gland; it was also present in normal apocrine but not eccrine sweat glands coils and ducts in skin. MFGM-gp 155 was present on the apical plasma membrane of the cells of lobules and terminal ducts, but not larger ducts of mammary gland, normal apocrine and eccrine sweat glands coils and ducts, or sebaceous glands in skin. Neither of the antibodies reacted with keratinocytes or melanocytes. Under similar conditions, the antibodies to MFGM-gp 70 reacted with Paget's cells of 8 of 8 cases of mammary disease and 6 of 8 cases of extramammary disease. By contrast, MFGM-gp 155 was localized in Paget's cells of 7 of 8 cases of mammary disease but none of the 8 cases of extramammary disease. The underlying tumour cells of infiltrating ductal breast carcinoma, where one was present, consistently showed reactivity with antibodies to MFGM-gp 70 and MFGM-gp 155. These findings lend additional support to the postulates that (a) Paget's cells in the breast originate in most cases from neoplastic mammary epithelial cells and (b) mammary and extramammary Paget cells, although morphologically similar, differ in expressing MFGM-gp 155.

More than a hundred years after the first pathological description of mammary and extramammary Paget's disease (Paget, 1874), researchers still seek to clarify both of the nature of the process and the cell of origin.

Several investigators using both light and electron-microscopical studies have proposed a variety of cell-types as the progenitors of Paget's cells, especially the extramammary variety (Ashikari *et al.*, 1970; Demopoulos, 1970; Ferenczy & Richart, 1972; Fetherston & Friedrich, 1972; Lee *et al.*, 1977; Mazonjian *et al.*, 1984; Medenica and Sahihi, 1972; Orr and Parish, 1962; Roth *et al.*, 1977; Sagebiel, 1969; Paone & Beker, 1981). The controversy over the histogenesis of Paget's cells has diverged into two main lines of opinion, *viz.* (a) migration of tumour cells from an underlying carcinoma or (b) developing as an independent *in situ* focus of malignant transformation in the epidermis. The former hypothesis has been widely supported especially in the case of Paget's disease of the nipple associated with an underlying invasive carcinoma of the breast. However, there persists a disagreement over the direction in which tumour cells migrate. Such disagreement is particularly evident with respect to Paget's disease of extramammary sites which is not always associated with an underlying neoplasm.

In an attempt to define the histogenesis of mammary and extramammary Paget's disease, the immunohistological staining patterns of antibodies to two immunologically and structurally distinct glycoproteins purified from human milk-fat-globule membrane (MFGM) were studied (Imam & Tokes, 1981; Imam *et al.*, 1981, 1982). The purified glycoproteins were designated MFGM-gp 70 and MFGM-gp 155 to indicate their origin and molecular weights (Imam & Tokes, 1981; Imam *et al.*, 1981, 1982, 1984, 1986). The patterns of distribution of these cell surface glycoproteins, the expression of which is related to cellular differentiation, shed light on the origin of neoplastic cells of Paget's disease of mammary and extramammary sites.

Correspondence: A. Imam.
Received 3 January 1988; and in revised form, 17 May 1988.

### Materials and methods

### *Materials*

Affinity-purified immunoglobulin G fraction of swine anti-rabbit immunoglobulin G and a rabbit peroxidase-antiperoxidase (PAP) complex were purchased from Cappel Laboratories, Malvern, PA. The chemical reagents used were of the highest purity available from Sigma Chemical Co., St. Louis, MO.

### *Purification of MFGM-gp 70 and MFGM-gp 155*

Two major glycoprotein components were purified to homogeneity from human milk-fat-globule membrane as described previously (Imam *et al.*, 1981, 1982, 1986). Each purified component yielded a single band under reducing conditions on SDS: polyacrylamide gel electrophoresis and has estimated molecular weights of 70,000 and 155,000 daltons (Imam *et al.*, 1981, 1982). They were designated MFGM-gp 70 and MFGM-gp 155. The physiochemical properties of these glycoproteins have been described (Imam *et al.*, 1984, 1986) and are summarized in Table I.

### *Production of antisera to MFGM-gp 70 and MFGM-155*

New Zealand white rabbits were immunized with the purified glycoprotein components, MFGM-gp 70 and MFGM-gp 155. The scheme for immunization was as described elsewhere (Imam *et al.*, 1981, 1982). The isolation of immunoglobulin G from the specific antisera and preimmune rabbit serum has also been as described previously (Imam *et al.*, 1986).

### *Preparation of tissue sections*

Formalin-fixed and paraffin embedded blocks of tissues of 8 cases each of mammary and extramammary Paget's disease were obtained from the files of the Division of Surgical Pathology, University of Southern California/Los Angeles

County Medical Center and the Good Samaritan Hospital, Los Angeles. The tissue blocks were sectioned at a thickness of 5 μm and several sections from each were stained with haematoxylin and eosin and examined to confirm the diagnosis.

*Immunoperoxidase staining method*

Formalin-fixed and paraffin-embedded blocks of human tissue (normal and neoplastic) were obtained from the surgical pathology files of University of Southern California/Los Angeles County Medical Center and the Good Samaritan Hospital, Los Angeles. For immunostaining, 5 μm tissue sections were cut from the paraffin-embedded blocks and stained by a standard peroxidase-antiperoxidase (PAP) technique as described previously (Imam & Taylor, 1985). The visual estimates of intensities of staining were graded as (−) absent, (1+) weak, (2+) moderate, or (3+) intense. To account for case to case variations in the degree of intensity of staining, any given tumor specimen was evaluated relative to a tissue section containing normal mammary epithelium which was scored as 3+ and it served as a positive control. The visual estimates of the percentage of cells stained were determined by examining at high magnification (400×) of 5 different and random fields on every tissue section. The mean of counts from the fields examined was used as the percentage of cells with staining.

For each experiment, 2 different controls were performed to ensure the specificity of the reaction. These included the use of immunoglobulin G fraction of specific antisera (i.e., anti MFGM-gp 70 and anti MFGM-gp 155) absorbed with their corresponding antigens (1 mg protein $ml^{-1}$) or preimmune rabbit serum.

### Results

*Immunohistological localization of MFGM-gp 70 and MFGM-gp 155*

The glycoproteins with reactivity to antibodies to MFGM-gp 70 and MFGM-gp 155 have been characterized and the findings reported (Imam *et al.*, 1984, 1986). A brief description of their physiochemical properties is summarized in Table I. The ability of these glycoproteins to retain their antigenic sites from denaturing, in formalin-fixed and paraffin-embedded tissue sections, has facilitated the study of their distributions by immunohistochemical methods under these conditions.

*Antibodies to MFGM-gp 70*

The binding characteristics of rabbit antiserum to MFGM-gp 70 to cells in normal mammary gland and skin are summarized in Table II. In normal breast, antibodies to MFGM-gp 70 reacted strongly with the luminal membrane of epithelial cells lining the ducts (Figure 1a) and lobules (not shown). In infiltrating ductal and lobular carcinomas of breast, the patterns of reactivity were markedly different, as shown by the presence of staining distributed both on the membrane and in cytoplasm, depending upon the morphological differentiation of the tumour cells (Imam *et al.*, 1984). The antibodies showed moderate to intense staining of the Paget's cells of all the 8 cases of mammary Paget's disease (Figure 1b, and Table III). These 8 cases of mammary Paget's disease tissue exhibited the presence of underlying infiltrating ductal carcinoma of the breast cell which showed consistent reactivity with the antibodies (Figure 1c). In 6 of 8 cases of extramammary Paget's disease, the tumour cells exhibited staining with the antibodies (Figures 1d, e and Table III). The proportion of cells stained within a tissue section varied from 60 to 90% of mammary and 60% to 80% of extramammary Paget's disease (Table III), revealing antigenic heterogeneity of the tumour cell population. Use of an equivalent amount of specific antibodies preabsorbed with MFGM-gp 70 or preimmune rabbit serum resulted in abolition of staining of the cells. Thus, it was concluded that binding of the antibodies to the cells represented specific reaction with their target epitopes and was not due to nonspecific binding of the immunoglobulin. Under these conditions, the normal cells in epidermis, lymphocytes, erythrocytes, endothelial cells and fat cells were consistently negative. Similar observations have been reported using the antibodies to study various grades of human mammary carcinomas (Imam *et al.*, 1984).

*Antibodies to MFGM-gp 155*

The binding properties of rabbit antibodies to MFGM-gp 155 to normal cells of mammary gland and skin are summarized in Table II. Antibodies to this glycoprotein can be used to quantitatively distinguish the luminal epithelial cells lining the lobules and terminal ducts (large arrow) from those lining large ducts (short arrows) in normal breast (Figure

**Table I** Physiochemical properties of MFGM-gp 70 and MFGM-gp 155

| | *MFGM-gp 70* | *MFGM-gp 155* |
|---|---|---|
| Molecular weight: | $70 \times 10^3$ daltons | $155 \times 10^3$ daltons |
| Isoelectric point: | 6.0 to 6.4 | 7.2 to 7.6 |
| Major amino acid: | Asparagine, glutamine serine | Asparagine, glutamine serine |
| Carbohydrate (% weight): | 13.5 | 21.00 |

**Table II** Immunohistological localization of MFGM-gp 70 and MFGM-gp 155 in luminal epithelial cells of normal mammary gland and skin tissue sections

| | *MFGM-gp 70* | | *MFGM-gp 155* | |
|---|---|---|---|---|
| *Tissue* | *Cell surface* | *Cytoplasm* | *Cell surface* | *Cytoplasm* |
| Mammary gland: | | | | |
| Large duct | Luminal strong | Luminal weak | Nonreactive | Nonreactive |
| Terminal duct | Luminal strong | Luminal weak | Luminal weak | Luminal weak |
| Lobule | Lumimal strong | Luminal weak | Luminal strong | Luminal weak |
| Skin: | | | | |
| Epidermis | Nonreactive | Nonreactive | Nonreactive | Nonreactive |
| Hair follicles | Nonreactive | Nonreactive | Nonreactive | Nonreactive |
| Eccrine coils and ducts | Nonreactive | Nonreactive | Nonreactive | Nonreactive |
| Apocrine coils and ducts | Luminal strong | Luminal weak | Nonreactive | Nonreactive |
| Sebaceous gland | Nonreactive | Weakly reactive | Nonreactive | Nonreactive |

**Figure 1** Indirect peroxide-antiperoxidase staining of formalin-fixed and paraffin-embedded tissue section with IgC fraction of polyclonal antibodies to MFGM-gp 70. Mayer's haemotoxylin counter stain; (a) Normal mammary epithelial cells lining the ducts showing reactivity mostly with their apical plasma membrane with the antibodies as indicated by arrows. Myoepithelial, fat and stomal cells are unreactive (original mag. × 312); (b) The antibodies reacted strongly with cytoplasm of Paget's cells of mammary gland (case 3). Note the absence of staining of keratinocytes and connective tissue elements (original mag. × 150); (c) Underlying unfiltrating ductal carcinoma cells from the above case showing both membrane and cytoplasmic staining with the antibodies (original mag. × 400); (d) The antibodies showed strong reactivity with the tumour cells of extramammary Paget's disease of the anus (case 6) (original mag. × 312), and (e) both membrane and cytoplasm of the tumour cells of extramammary Paget's disease of groin (case 8) exhibiting intense staining with the antibodies (original mag. × 150).

2a). The expression of the antigen was maintained in morphologically well and poorly differentiated infiltrating ductal and lobular carcinomas of breast. However, the expression of the antigen was much denser on the malignant cells of lobular compared with infiltrating ductal carcinomas of comparable morphological differentiation (Imam *et al.*, 1986). The antiserum reacted with variable degree of intensity to 60% to 85% of Paget's cells of 7 of 8 cases of mammary (Figure 2b) and none of the 8 cases of extramammary Paget's disease (Table IV). Thus, like antiserum to MFGM-gp 70, the antiserum to MFGM-gp 155 showed a similar pattern of reactivity to tumour cells of mammary Paget's disease (Figure 1b, 2b), but differed in exhibiting no reactivity with tumour cells of extramammary Paget's disease (Table IV). The underlying infiltrating ductal carcinoma cells, when present in association with mammary Paget's disease, showed consistent reactivity with the antibodies with variable intensity (Figure 2c). There was no detectable reactivity with sebaceous or eccrine glands, squamous epithelial cells, lymphocytes, erythrocytes, enthothelial and fat cells. In control experiments, use of an equivalent amount of specific antibodies preabsorbed with MFGM-gp 155 or

**Figure 2** Indirect peroxidase-antiperoxidase staining of formalin-fixed and paraffin embedded tissue sections with IgG fraction of polyclonal antibodies to MFGM-gp 155. Mayer's hemotoxylin counter stain; (a) Normal breast luminal mammary epithelial cells of terminal ductules in lobules showing reactivity predominantly on the apical membrane with the antibodies as indicated by long arrows. Note the absence of reactivity with the luminal epithelium lining the large ducts as indicated by short arrows, myoepithelial and stomal cells (original mag. x 400); (b) The antibodies showing reactivity with the cytoplasm of the tumour cells of mammary Paget's disease (case 5). Even a single tumour cell in epidermis can be visualized as indicated by arrows. Note the absence of staining of keratinocytes and elements of supporting stroma (original mag. x 150); (c) Underlying infiltrating ductual carcinoma cells from the above case exhibiting both membrane and cytoplasmic reactivity with the antibodies (original mag. x 150).

preimmune rabbit serum resulted in the abolition of staining of the cells, demonstrating the specificity of reactivity.

### Discussion

Mammary and extramammary Paget's cells are now generally accepted to represent intraepidermal carcinoma cells of glandular differentiation which are usually, but not always, associated with an *in situ* or infiltrating adenocarcinoma within the underlying ducts or adnexal structures. Supportive immunohistochemical evidence for the glandular nature of Paget's cells comes from Bussolati and Pich (1975) who have demonstrated the presence of casein in mammary and extramammary Paget cells, and from several studies demonstrating immunoreactivity of mammary and extramammary Pagets cells with cytokeratin antibodies that selectively react with glandular epithelial cells (Kariniemi *et al.*, 1985, Hamm & Vroon, 1986 & Ordonez *et al.*, 1987). In addition, Nadji *et al.*, (1982) have shown similar patterns of immunoreactivity for carcinoembryonic antigen (CEA) within mammary and extramammary Paget's cells, and Mazoujian *et al.* (1984) demonstrated immunoperoxidase localization of GCDFP-15 in extramammary Paget cells in some cases. More recently, Kariniemi *et al.* (1984), Kirkham *et al.* (1985) and Vanstapel *et al.* (1984) have demonstrated the expression of antigens recognized by their own antibodies to human milk-fat-globule membrane in tumour cells of both mammary and extramammary Paget's diseases.

In the present study, polyclonal antibodies to two immunologically distinct glycoproteins purified from human milk-fat-globule membrane (MFGM) (Imam *et al.*, 1981, 1982) were used to study the specificity and patterns of reactivity in tumour cells of mammary and extramammary Paget's disease. These glycoproteins are immunologically and biochemically distinct from each other and are unrelated to milk whey proteins, serum proteins, blood group antigens, lymphocyte antigens, keratins, carcinoembryonic antigen and mouse mammary tumour virus glycoproteins (Imam *et al.*, 1981, 1982).

The finding of reactivity of antibodies against MFGM-gp 70 to Paget's cells of both mammary and extramammary Paget's disease confirms previous studies that Paget's cells show differentiation features in common with glandular epithelium (Table III). Furthermore, MFGM-gp 70 and MFGM-gp 155 positivity in the tumour cells of mammary Paget's disease demonstrates the phenotypic similarity between mammary Paget's cells and underlying mammary carcinoma, since both MFGM-gp 70 and MFGM-gp 155 are also commonly present in normal breast and mammary carcinoma cells (Tables II–IV).

Interestingly, while 7 of 8 cases of mammary Paget's disease expressed MFGM-gp 155, all 8 cases of extramammary Paget's disease were nonreactive for the antigen under the conditions employed in this study. The possibility that formalin-fixation may alter MFGM-gp 155 configuration in extramammary Paget's cells in tissue reactions, leading to the negative staining seems unlikely as tissues other than breast fixed under the same conditions showed consistently strong reactivity with the antibodies to MFGM-gp 155 (Imam *et al.*, 1986). MFGM-gp 155 is restricted in its tissue localization to mammary lobules and terminal ducts, and absent from apocrine and eccrine glandular epithelia. While its expression in mammary Paget's cells confirms a phenotypic similarity with mammary epithelia, its complete lack of expression in all cases of extramammary Paget's disease likewise demonstrates a phenotypic dissimilarity between extramammary Paget's cells and mammary epithelia. This difference in MFGM-gp 155 antigenic expression thus constitutes evidence that mammary and extramammary Paget's cells, although morphologically similar, show differences in phenotype and are probably distinct developmentally. Further supportive evidence of their phenotypic distinction is provided by apparent differences in cytokeratin proteins between Paget's cells of the vulva and breast (Ordonez *et al.*, 1987). Also, evidence of a histogenetic difference between mammary and extramammary Paget's

**Table III** Immunohistological localization of MFGM-gp 70 in neoplastic cells of mammary and extramammary Paget's disease

| *Case* | *Mammary Paget's disease (site)* | *Intensity staining* | *% of cells stained* | *Extramammary Paget's disease (site)* | *Intensity staining* | *% of cells stained* |
|---|---|---|---|---|---|---|
| 1 | Nipple | 3+ | 85 | Vulva | 2+ | 75 |
| 2 | Nipple | 2+ | 60 | Vulva | 3+ | 60 |
| 3 | Nipple | 2+ | 90 | Vulva | – | 0 |
| 4 | Nipple | 2+ | 75 | Leg | – | 0 |
| 5 | Nipple | 2+ | 60 | Anus | 1+ | 60 |
| 6 | Nipple | 3+ | 60 | Anus | 2+ | 80 |
| 7 | Nipple | 2+ | 90 | Groin | 1+ | 75 |
| 8 | Nipple | 3+ | 60 | Groin | 1+ | 80 |

[a]Sections were recorded for the intensity of staining on scale from – to 3+. Absence of staining: –; weak staining: 1+; moderate staining: 2+; intense staining: 3+.

**Table 4** Immunohistological localization of MFGM-gp 155 in neoplastic cells of mammary and extramammary Paget's disease

| *Case* | *Mammary Paget's disease (site)* | *Intensity staining* | *% of cells stained* | *Extramammary Paget's disease (site)* | *Intensity staining* | *% of cells stained* |
|---|---|---|---|---|---|---|
| 1 | Nipple | 2+ | 60 | Vulva | — | 0 |
| 2 | Nipple | 1+ | 70 | Vulva | — | 0 |
| 3 | Nipple | 2+ | 60 | Vulva | — | 0 |
| 4 | Nipple | — | 0 | Leg | — | 0 |
| 5 | Nipple | 2+ | 85 | Anus | — | 0 |
| 6 | Nipple | 2+ | 70 | Anus | — | 0 |
| 7 | Nipple | 1+ | 60 | Groin | — | 0 |
| 8 | Nipple | 1+ | 75 | Groin | — | 0 |

[a]Sections were recorded for the intensity of staining on scale from – to 3+. Absence of staining: –; weak staining: 1+; moderate staining: 2+; intense staining: 3+.

cells is provided by the recent description of intraepidermal putative aberrant embryonic cells of sweat gland derivation in two young siblings (Kuo *et al.*, 1987). It was postulated that such cells may represent the benign counterpart or precursor cell of extramammary Paget's disease.

An apocrine derivation for extramammary Paget's disease has been previously hypothesized, based upon its expression of gross cystic disease fluid protein-15 (Mazoujian *et al.*, 1984). However, GCDFP-15, initially reported to be localized within apocrine but not eccrine glands, has subsequently been shown to be expressed by both apocrine as well as eccrine glands (Ordonez *et al.*, 1987). Our findings of MFGM-gp 70 in apocrine glandular epithelium and the lack thereof in eccrine glandular epithelium provide evidence that MFGM-gp 70 may function as a presumptive marker of apocrine differentiation. Therefore, the finding that extramammary Paget's cells, in the majority of cases of extramammary Paget's disease, express MFGM-gp 70 immunoreactivity suggests that extramammary Paget's cells derive from or differentiate along the line of apocrine glandular epithelium. The lack of MFGM-gp 155 expression by extramammary Paget's cells provides supportive evidence because apocrine, as well as eccrine, glands also lack MFGM-gp 155 expression. However, we cannot entirely rule out the possibility that the two cases of extramammary Paget's disease negative for MFGM-gp 70 are of eccrine derivation. Isolated case reports suggest that such an eccrine line of differentiation may occur in a few cases (de Blois *et al.*, 1984).

In summary, the expression of MFGM-gp 155 is quite specific in its localization to breast lobules and terminal ducts and is not found in apocrine and eccrine glandular, and squamous epithelial cells; it is expressed in mammary Paget's cells but not in Paget's cells of extramammary disease. By contrast, MFGM-gp 70 appears to be present in both breast and apocrine cells, but not eccrine and squamous epithelial cells; it is present in most cases of Paget's disease, whether mammary or extramammary in situation. These results suggest that Paget's cells in the breast originate, in most cases, from neoplastic mammary epithelial cells, and that extramammary Paget's cells are of apocrine origin as revealed by the immunohistological studies. Furthermore, mammary and extramammary Paget's cells show striking differences with respect to the expression of MFGM-gp 155, suggesting that the two types of Paget's cells are phenotypically different.

### References

ASHIKARI, R., PARK, K., HUVOS, A.G. & URBAN, J.A. (1970). Paget's disease of the breast. *Cancer*, **26**, 680.

BUSSOLATI, G. & PICH, A. (1975). Mammary and extramammary Paget's disease. An immunocytochemical study. *Am. J. Pathol.*, **80**, 117.

DE BLOIS, G.G., PATTERSON, J.W. & HUNTER, S.B. (1984). Extramammary Paget's disease: Arising in knee region in association with sweat gland carcinoma. *Arch. Pathol. Lab. Med.*, **108**, 713.

DEMOPOULOS, R.I. (1970). Fine structure of the mammary Paget's cell. *Cancer*, **27**, 1202.

FERENCZY, A. & RICHART, R.M. (1972). Ultrastructure of perianal Paget's disease. *Cancer*, **29**, 1141.

FETHERSTON, W.C. & FRIEDRICH, E.C. (1972). The origin and significance of vulvar Paget's disease. *Obstet Gynecol.*, **39**, 735.

HAMM, H. & VROON, T.M. (1986). Extramammary Paget's cells: Further evidence of sweat gland derivation. *J. Am. Acad. Dermatol.*, **15**, 1275.

IMAM, A. & TOKES, Z.A. (1981). Immunoperoxidase localization of a glycoprotein on plasma membrane of secretory epithelium from human breast. *J. Histochem. Cytochem.*, **29**, 581.

IMAM, A., LAURENCE, D.J.R. & NEVILLE, A.M. (1981). Isolation and characterization of a major glycoprotein from milk-fat-globule membranes of human breast milk. *Biochem. J.*, **193**, 47.

IMAM, A., LAURENCE, D.J.R. & NEVILLE, A.M. (1982). Isolation and characterization of two individual glycoproteins from human milk-fat-globule membranes. *Biochem. J.*, **209**, 37.

IMAM. A., TAYLOR, C.R. & TOKES, Z.A. (1984). Immunohistochemical study of the expression of human milk-fat-globule membrane, glycoprotein 70. *Cancer Res.*, **44**, 2016.

IMAM, A., TAYLOR, C.R. (1985). Application of immunohistochemical methods in the diagnosis of malignant disease. *Cancer Invest.*, **3**, 339.

IMAM, A. (1986). Investigation into the asymmetric distribution of proteins in human milk-fat-globule-membrane. *J. Biochem.*, **99**, 143.

IMAM, A., DRUSHELLA, M.M., TAYLOR, C.R. & TOKES, Z.A. (1986). Preferential expression of a *Mr* 155,000 milk-fat-globule membrane glycoprotein on luminal epithelium of globules in human breast. *Cancer Res.*, **46**, 6374.

KARINIEMI, A.-L., FORSMAN, L., WAHLSTROM, T., VESTERINEN, E. & ANDERSSON, L. (1984). Expression of differentiation antigens in mammary and extramammary Paget's disease. *Br. J. Dermatol.*, **110**, 203.

KARINIEMI, A.-L., RAMAEKERS, F., LEHTO, V.-P. & VIRTANEN, I. (1985). Paget cells express cytokeratins typical of glandular epithelia. *Br. J. Dermatol.*, **112**, 179.

KIRKHAM, N., BERRY, N., JONES, D.B. & TAYLOR-PAPADIMITRION, J., (1985). Paget's disease of the nipple: Immunohistochemical localization of milk-fat-globule membrane antigens. *Cancer*, **55**, 1510.

KUO, T.-T., CHAN, H.-L. HSUEH, A. (1987). Clear cell papulosis of the skin: A new entity with histogenetic implications for cutaneous Paget's disease. *Am. J. Surg. Pathol.*, **11**, 827

LEE, S.C., ROTH, L.M., EHRLICH, C. & HALL, J.A. (1977). Extramammary Paget's disease of the vulva. *Cancer*, **39**, 2540.

MAZOUJIAN, G., PINKUS, G.S. & HAAGENSEN, D.E. (1984). Extramammary Paget's disease – Evidence for an apocrine origin. *Am. J. Surg., Pathol.*, **8**, 43.

MEDENICA, M., SAHIHI, T. (1972). Ultrastructure study of a case of extramammary Paget's disease of the vulva. *Arch. Dermatol.*, **105**, 236.

NADJI, M., MORALES, A.R., GIRTANNER, R.E.., ZIEGES-WEISSMAN, J. & PENNEYS, N.S. (1982). Paget's disease of the skin. A unifying concept of histogenesis. *Cancer*, **50**, 2203.

ORDONEZ, N.H., AWALT, Z., MACKAY, B. (1987). Mammary and extramammary Paget's disease. An immunocytochemical and ultrastructure study. *Cancer*, **59**, 1173.

ORR, J.W. & PARISH, D.J. (1962). The nature of the nipple changes in Paget's disease. *J. Pathol. Bacteriol.*, **84**, 201.

PAGET, J. (1874). On disease of mammary areola preceding cancer of mammary gland. *St Bartholomew Hospital Rep.*, **10**, 87.

PAONE, J.F. & BEKER, R.R. (1981). Pathogenesis and treatment of Paget's disease of the breast. *Cancer*, **48**, 825.

ROTH, L.M., LEE, S.C. & EHRLICH, C.E. (1977). Paget's disease of the vulva: A histologic study of five cases including ultrastructural observation and review of the literature. *Am. J. Surg. Pathol.*, **1**, 193.

SAGEBIEL, R.W. (1969). Ultrastructure observations on epidermal cells in Paget's diseases of the breast. *Am. J. Pathol.*, **57**, 49.

VANSTAPEL, M.-V., GATTER, K.C., DEWOLF-PEETERS, C., MILLARD, P.R., DESMET, V.J. & MASON, D.Y. (1984). Immunohistochemical study of mammary and extramammary Paget's disease. *Histopathology*, **8**, 1013.